# Dengue in a crowded megacity: Lessons learnt from 2019 outbreak in Dhaka, Bangladesh

**DOI:** 10.1371/journal.pntd.0008349

**Published:** 2020-08-20

**Authors:** Mohammad Sorowar Hossain, Mahbubul H. Siddiqee, Umme Ruman Siddiqi, Enayetur Raheem, Rokeya Akter, Wenbiao Hu

**Affiliations:** 1 Biomedical Research Foundation, Dhaka, Bangladesh; 2 School of Environment and Life Sciences, Independent University, Bangladesh (IUB), Dhaka, Bangladesh; 3 Department Mathematics and Natural Sciences (MNS), BRAC University, Mohakhali, Dhaka, Bangladesh; 4 Zoonotic Disease Control Program, Directorate General of Health Services, Dhaka, Bangladesh; 5 School of Public Health and Social Work, Queensland University of Technology, Brisbane, Australia; USDA-ARS Center for Medical Agricultural and Veterinary Entomology, UNITED STATES

## Background

Dengue is a mosquito-borne viral disease commonly reported in the tropical regions of the world. The presence of two mosquito vectors (*Aedes aegypti* is highly urban, while *A*. *albopictus* is less urban) throughout the year makes dengue fever an endemic disease in a number of countries. Among the predicting variables, a rise of temperature and rainfall have shown to be associated with the number of dengue cases [1]. While relatively less emphasized compared to the climatic factors, mass movement is particularly important during large-scale outbreaks. This article presents a case based on the available data from the 2019 outbreak in Bangladesh, where the dengue fever was initially concentrated in Dhaka, the capital city.

Dhaka is one of the most crowded megacities in the world, with over 19 million people distributed over 453 square km (spatial density of 41,000/square km) [2]. It is a hub of all key administrative, educational, and industrial activities in Bangladesh. Hence, a considerable proportion of the Bangladeshi population prefers to live in the city for better income, quality education, and better facilities overall. The 2019 dengue outbreak in Dhaka was the most intense since the emergence of dengue outbreaks in late the 1990s in Bangladesh [3].

According to Directorate General of Health Services (DGHS), Bangladesh, a total of 100,201 confirmed dengue cases were admitted in hospitals between January and December 2019; 51,179 cases were reported in Dhaka city and 49,022 across the rest of Bangladesh [4]. This implies a 10-fold increase in hospitalized cases compared with the largest outbreak before 2019. Since the first major outbreak in 2000, all four serotypes (DEN-1–4) were reported in Dhaka city until 2003, a with higher prevalence of DEN-3 serotype. After a hiatus (2013–2016), DEN-3 serotype re-emerged in 2017, and this serotype has been reported to be the most frequently identified during the 2019 outbreak [5,6]. Anecdotal evidence from medical practitioners suggests that dengue patients manifested with a spectrum of atypical symptoms in the 2019 outbreak, which can be related to serodiversity of the viral strains.

A recently published nationwide seroprevalence study (2014–2015) showed that 24% of participants had past history of dengue infection in Bangladesh [7]. Seropositivity of the infected individuals was largely confined to three big cities, namely Dhaka, Chittagong, and Khulna, indicating that circulation of dengue virus was not high in the semi-urban and rural areas of Bangladesh [7]. Notably, all major dengue outbreaks in Bangladesh showed a tendency to remain confined mostly in Dhaka city [7], and the 2019 outbreak showed the same trend until it reached its peak in August (having more than 50% of the total case reported from January to December) [3]. Then gradually, unlike all the previous outbreaks in Bangladesh, the 2019 outbreak started to spread across the country. We hypothesize that mass movement of people who are already infected with the virus can accelerate the spreading of the disease to areas where the burden of dengue fever was otherwise low.

## Methods

Daily dengue case data were collected from the DGHS, while daily rainfall and temperature data during 2012–2019 were obtained from the Bangladesh Meteorological Department. Population consensus (2011) was obtained from the Bangladesh Bureau of Statistics. Time series analysis was performed to see the patterns of temperature and rainfall during and around the outbreak compared to the historical averages. A simple autoregressive integrated moving average (ARIMA) model (1,0,0) was developed after controlling time series auto-correlation to explore if there was sufficient structure in the time series of dengue incidences to be explained by human movement. A district-level difference map between the incidence of one week after Eid and one week before Eid was created and visualized using ArcMap (version 10.6).

## Results

**Fig 1:**
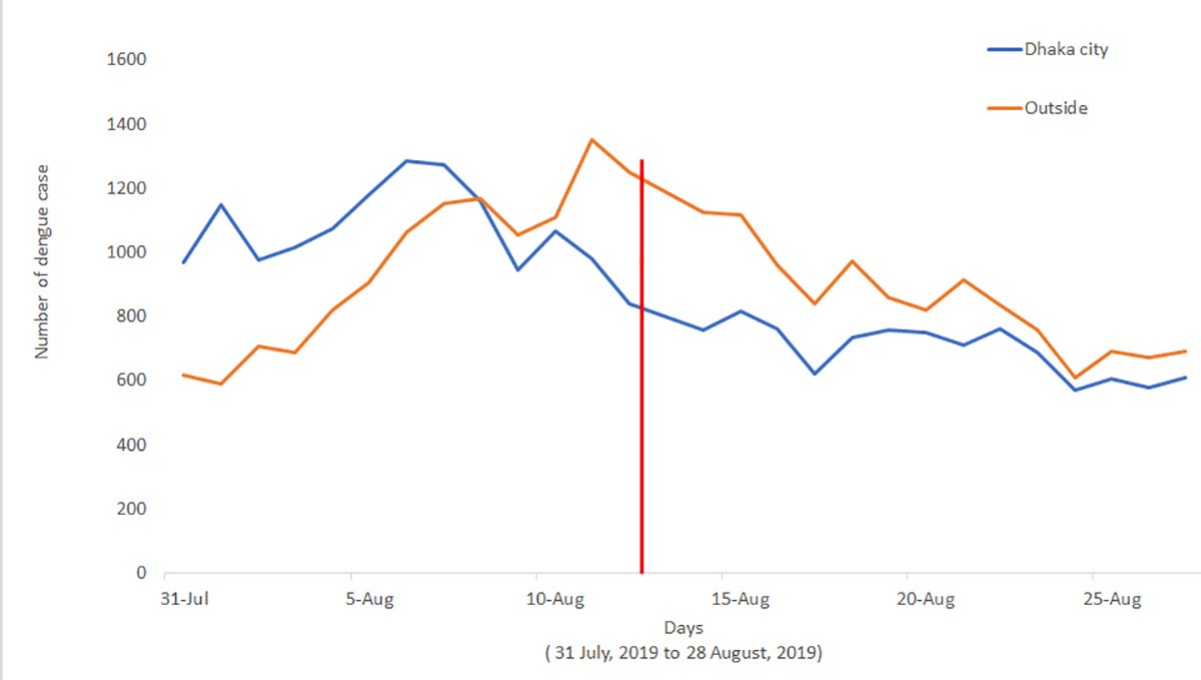
Daily time series distribution of dengue during the outbreak, 2019. Red line shows the Eid day during the outbreak.

**Fig 2:**
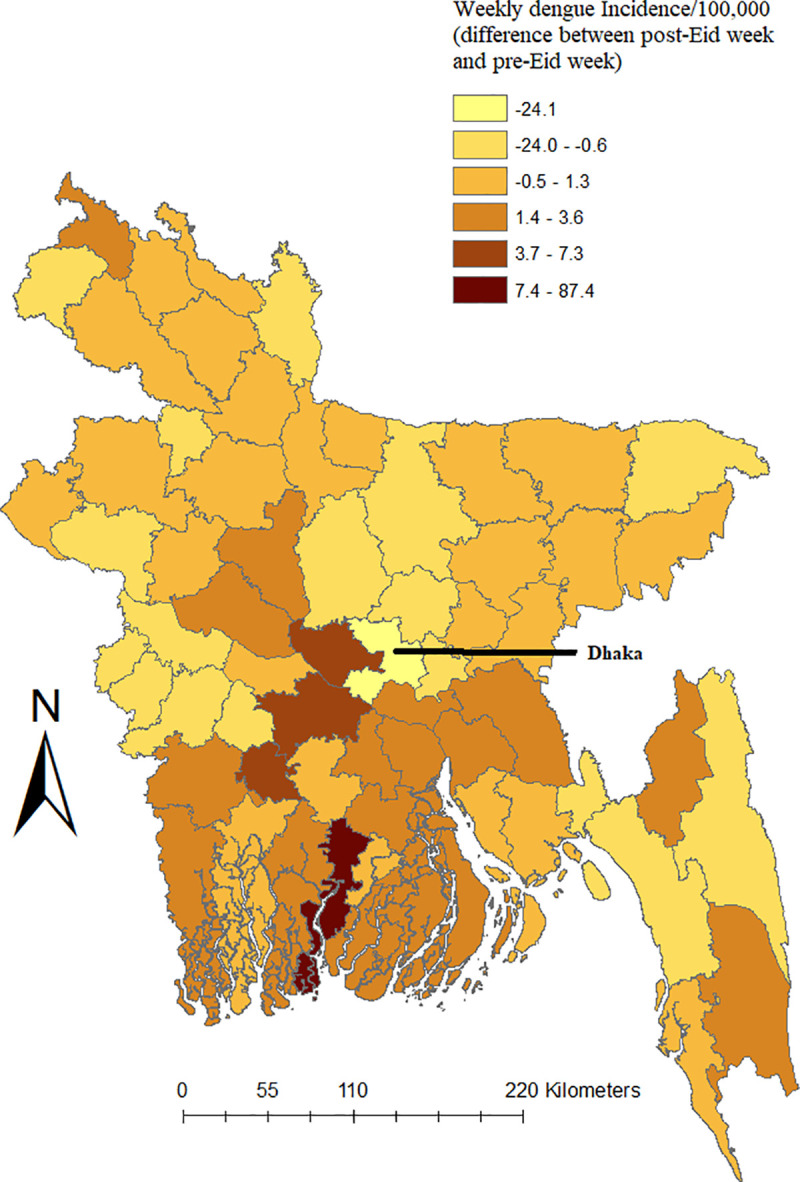
Spatial variation of differences of weekly dengue incidence between post-Eid and pre-Eid week in different districts of Bangladesh. Positive and negative values in the legend represent increase and decrease of weekly dengue incidence, respectively. To calculate weekly incidence, total number of dengue cases for the week for each district was divided by the total population of the respective district and multiplied by 100,000.

In July 2019 (prior to Eid), over 81% of all dengue cases (16,253) were reported within the Dhaka city [4]. Before Eid exodus (first week of August 2019), an interesting pattern of frequency of dengue case reporting was observed: during the week prior to Eid, 57% (*n* = 7,952) of all the cases were reported inside Dhaka compared to 43% (*n* = 5,927) throughout the rest of Bangladesh, indicating the outbreak was mostly localized up until that point (Table 1, Fig 1). However, this pattern started to reverse during the Eid week, when the number of cases outside Dhaka started to rise to nearly 55% (*n* = 7,663) of the total cases. The same trend sustained after Eid weeks. While the incidence of dengue cases decreased sharply in Dhaka city after the Eid week (as much as about 24 times compared to pre-Eid week), a net increase (around 4–7-fold) was observed in some districts of Bangladesh, including Narail, Pirojpur, Manikgonj, and Faridpur (Fig 2). Time series cross-correlation and autoregressive model showed that the dengue within Dhaka city appeared to play a significant role in the transmission of dengue outside of Dhaka (S1 Fig, S1 Table).

**Table 1:** Distribution of weekly dengue cases in and outside Dhaka, Bangladesh, during the festive month and the month after festivity.

**August, with Eid festivity**				
**Areas affected**	**Week 1** **(Pre-Eid)**	**Week 2** **(Eid Week)**	**Week 3** **(Post-Eid)**	**Week 3** **(Post-Eid)**
Dhaka city	7,952 (57.3%)	6,351 (45.3%)	5,149 (44.3%)	4,363 (47.2%)
Outside Dhaka city	5,927 (42.7%)	7,663 (54.7%)	6,484 (55.7%)	4,878 (52.8%)
Total	13,879	14,014	11,633	9,241
**September, without Eid festivity**				
Dhaka city	2,379 (41.8%)	1,751 (36.3%)	1,188(29.5%)	838 (29.2%)
Outside Dhaka city	3,313 (58.2%)	3,072 (63.7%)	2,844(70.5%)	2,028 (70.8%)
Total	5,692	4,823	4,032	2,866

## Discussion

In Bangladesh, all the dengue outbreaks since 2000 were primarily confined to Dhaka city [7]. However, it is expected to spread in other areas because of the presence of *Aedes* mosquitoes throughout Bangladesh [6]. Based on a survey conducted in 2014–2015, both *A*. *aegypti* and *A*. *albopictus* were found in Dhaka city and in other urban areas. While the prevalence of *A*. *aegypti* was higher in urban areas, *A*. *albopictus* was more abundant in rural areas across Bangladesh [6]. Therefore, any scenario that increases migration of dengue patients and the carriers from Dhaka to other regions could have the virus picked up by a new host (i.e., *A*. *albopictus*).

We observed that the timing of changing the spatial pattern coincided with the Eid. Just before this time, nearly half of the population in Dhaka (over 10 million) leave the city and travel across the country, usually within a week (to reunite with their family members) [8]. Hence, based on the data analyzed from the 2019 outbreak, it can be hypothesized that the mass mobility during Eid might have contributed to the spread of the outbreak. In this regard, the potential effects of climate factors on mosquito breeding were also considered. Changes in climatic conditions could be associated with dynamic changes in the temporal distribution of dengue. However, Bangladesh is a relatively small flat country, and with Dhaka being geographically positioned in the middle, climatic factors like temperature and rainfall usually do not vary significantly [9]. Therefore, while high temperatures during August 2019 might have influenced the largest outbreak in Dhaka (potentially by influencing the extrinsic incubation period [EIP] in the mosquitoes), a similar trend would have been seen for both Dhaka and outside Dhaka as the similar high temperature was reported all over Bangladesh. However, a delayed rise of incidence in dengue fever outside Dhaka at the time when incidence within Dhaka was decreasing suggests that the temperature was most likely not the key factor in this case. Instead, the geographic expansion of dengue in new populations was more likely facilitated by the travel of infected individuals to non-endemic areas.

Previous studies showed that transport vehicles could disperse *Aedes* mosquitoes within a country [10]. Lack of nationwide epidemiological studies made it difficult to verify whether the rise in dengue cases outside Dhaka was due to a large number of infected individuals traveling as asymptomatic carriers. However, a number of investigative reports published in the national daily newspapers could provide an essential clue in this regard; stories of clustered infections among people not having a history of traveling to Dhaka during one month before onset was frequent. This eventually suggests that transmission of dengue virus (or both virus and mosquito) during the mass migration before the Eid was likely the most significant factor behind the geographical distribution of the diseases.

From a public health standpoint, such mass mobility events (e.g., Eid exodus, Hajj pilgrimage, migration during the Chinese New Year) can sometimes be reasons for major concern if their location and timing coincide with major outbreak [11]. Such unexpected coincidence could facilitate outbreaks (especially with highly contagious viruses) morphing into an epidemic or even pandemic (depending on other factors like ease of transmission and favorability of climate factors). The ongoing outbreak of coronavirus in Wuhan, China, can be a recent example of this. Transmission and spread of infectious diseases, including dengue and other *Aedes* mosquito-borne diseases (chikungunya and Zika), especially during an outbreak can therefore present an immense challenge for public health safety, as there is no vaccine against dengue. This is especially true for the countries that have high population density and have a limited capacity to manage an increasing number of patients beyond the urban areas where the healthcare facilities are relatively well equipped. Therefore, efforts taken by the regulatory authorities to restrain the disease need to take this mass migration–associated risk into account. Public health preparedness can be an effective tool to reduce mortality and morbidity. In this regard, alongside conventional measures (e.g., the establishment of a comprehensive disease surveillance system, integrated vector control and management through community awareness, implementation of early diagnosis, prompt treatment, and effective patient referral protocol through trained caregivers at all levels of healthcare facilities), unconventional measures, particularly limiting or prohibiting mass migration (depending on the level of risk), could be considered. Attempts at conducting risk assessment based on early warning signs therefore need to incorporate social factors and mass movement events along with the climate factors.

## Supporting information

S1 Table ARIMA model (1,0,0) after controlling time series auto-correlation. ARIMA, autoregressive integrated moving average.
(DOCX)

S1 Fig Cross-correlation analysis of dengue within and outside of Dhaka. (DOCX)

S2 Fig A time series analysis of average monthly rainfall in Dhaka during 2012–2019. (DOCX)
