# Supplementary material for: Dengue in a crowded megacity: Lessons learnt from 2019 outbreak in Dhaka, Bangladesh
Source: PLoS Negl Trop Dis. 2020 Aug 20;14(8):e0008349. doi: 10.1371/journal.pntd.0008349 (PMC7444497; doi:10.1371/journal.pntd.0008349)
Supplement: S1 Table — ARIMA, autoregressive integrated moving average. (DOCX) [file pntd.0008349.s001.docx]

**S1 Table 1:** Autoregressive integrated moving average (ARIMA) model (1,0,0) after controlling time series auto-correlation.

| **ARIMA Model Parameters** | | | | | | | | |
| --- | --- | --- | --- | --- | --- | --- | --- | --- |
|  | | | | | Estimate | SE | t | Sig. |
| Dengue outside Dhaka |  |  | Constant | | 23.133 | 6.475 | 3.573 | .001 |
|  |  |  | AR | Lag 1 | .938 | .064 | 14.583 | .000 |
|  | EID (yes/no) |  |  | Lag 0 | -7.776 | 2.614 | -2.974 | .006 |
|  | Dengue within Dhaka |  |  | Lag 0 | .010 | .004 | 2.254 | .033 |
