# Supplementary material for: Dengue in a crowded megacity: Lessons learnt from 2019 outbreak in Dhaka, Bangladesh
Source: PLoS Negl Trop Dis. 2020 Aug 20;14(8):e0008349. doi: 10.1371/journal.pntd.0008349 (PMC7444497; doi:10.1371/journal.pntd.0008349)
Supplement: S1 Fig — (DOCX) [file pntd.0008349.s002.docx]

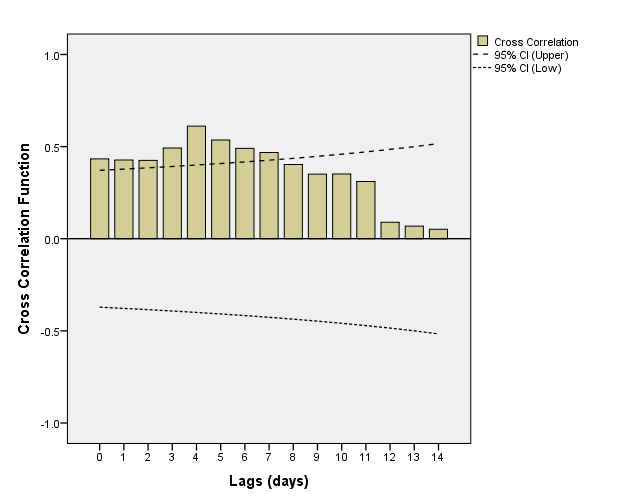


**S1 Fig:** Cross-correlation analysis of dengue within and outside of Dhaka. Cross-correlation analysis showed that the dengue cases outside of Dhaka at a lag of 0 – 7 days were significantly associated with the dengue within Dhaka. An autoregressive integrated moving average (ARIMA) model (1,0,0) was developed after controlling time series auto-correlation, which also confirmed the relationship between dengue outside Dhaka and dengue within Dhaka. The results showed that the dengue within Dhaka (β= 0.01, p = 0.033). and EID (β= -7.776, p = 0.006) appeared to play a significant role in the transmission of dengue outside Dhaka (Suppl. Table 1).
