# Supplementary material for: Dengue in a crowded megacity: Lessons learnt from 2019 outbreak in Dhaka, Bangladesh
Source: PLoS Negl Trop Dis. 2020 Aug 20;14(8):e0008349. doi: 10.1371/journal.pntd.0008349 (PMC7444497; doi:10.1371/journal.pntd.0008349)
Supplement: S2 Fig — (DOCX) [file pntd.0008349.s003.docx]

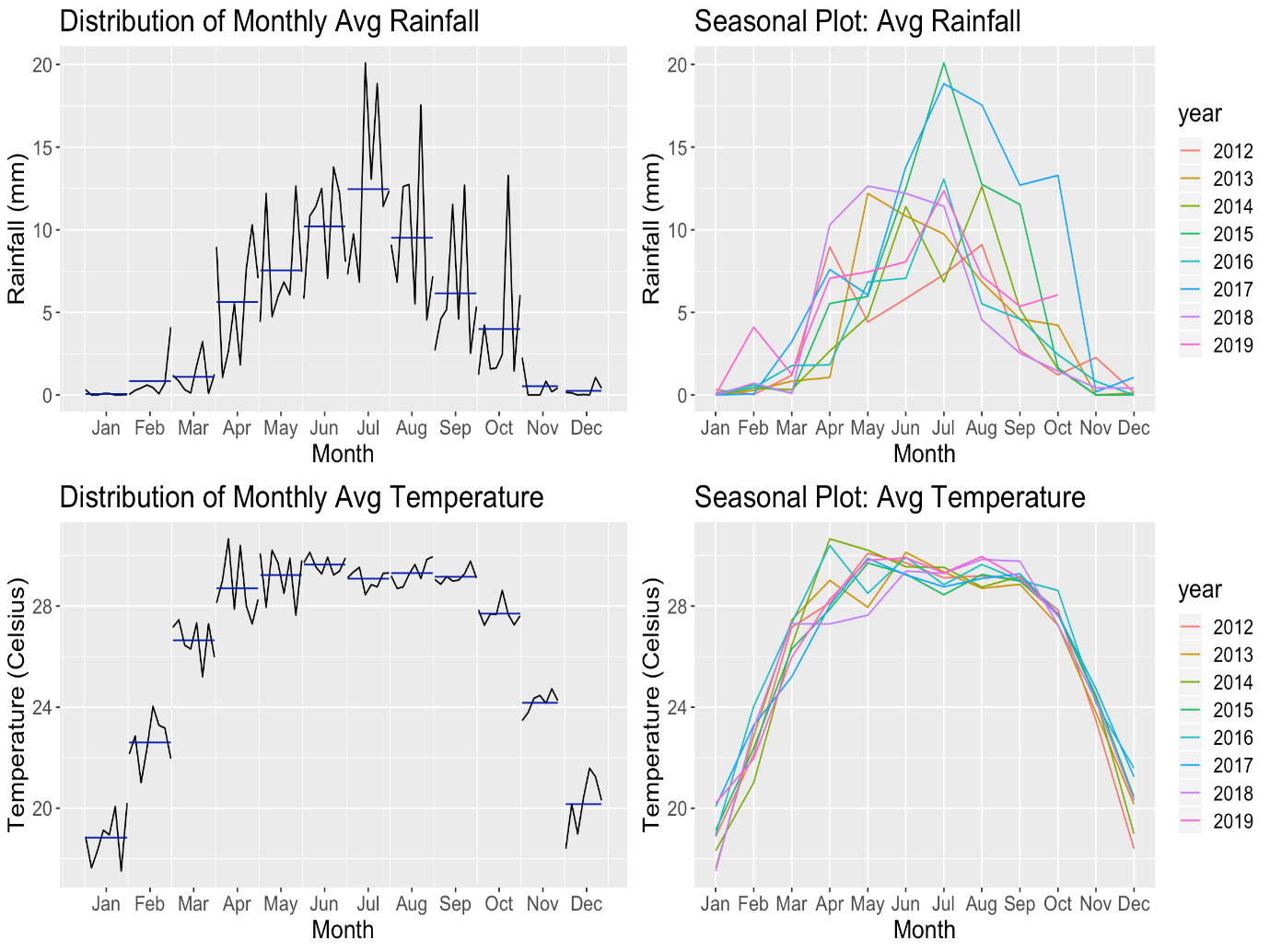


**S1 Fig 2:** A time series analysis of average monthly rainfall in Dhaka during 2012-2019. *Top left panel:* shows the historical average monthly rainfall for August was 9.51 mm whereas in 2019 it was slightly below average at 7.19 mm. *Top right panel:* shows seasonal plot of average rainfall over the last 8 years. The average rainfall in August 2019 (peak of dengue outbreak) was consistent with the historical pattern. Similarly, the historical average temperature for August was 29.3 degree Celsius compared to 30 (degree Celsius) observe in August 2019. The seasonal plots for rainfall and temperature (*right panels*) do not show any unusual pattern. It is to be noted that August 2019 appears to be the hottest August in terms of average temperature in the past 8 years.
